# RNApysoforms: fast rendering interactive visualization of RNA isoform structure and expression in Python

**DOI:** 10.1093/bioadv/vbaf057

**Published:** 2025-03-14

**Authors:** Bernardo Aguzzoli Heberle, Madeline L Page, Emil K Gustavsson, Mina Ryten, Mark T W Ebbert

**Affiliations:** Sanders-Brown Center on Aging, University of Kentucky, Lexington, KY, 40536-0298, United States; Department of Neuroscience, College of Medicine, University of Kentucky, Lexington, KY, 40536-0298, United States; Sanders-Brown Center on Aging, University of Kentucky, Lexington, KY, 40536-0298, United States; Department of Genetics and Genomic Medicine, Great Ormond Street Institute of Child Health, University College London, London, WC1N 1EH, United Kingdom; Aligning Science Across Parkinson’s Collaborative Research Network, Chevy Chase, MD, 20815, United States; Department of Genetics and Genomic Medicine, Great Ormond Street Institute of Child Health, University College London, London, WC1N 1EH, United Kingdom; Aligning Science Across Parkinson’s Collaborative Research Network, Chevy Chase, MD, 20815, United States; UK Dementia Research Institute at The University of Cambridge, Cambridge, CB2 0AH, United Kingdom; Department of Clinical Neurosciences, School of Clinical Medicine, University of Cambridge, Cambridge, CB2 0SP, United Kingdom; Sanders-Brown Center on Aging, University of Kentucky, Lexington, KY, 40536-0298, United States; Department of Neuroscience, College of Medicine, University of Kentucky, Lexington, KY, 40536-0298, United States; Division of Biomedical Informatics, Internal Medicine, College of Medicine, University of Kentucky, Lexington, KY, 40536, United States

## Abstract

**Summary:**

Alternative splicing generates multiple RNA isoforms from a single gene, enriching genetic diversity and impacting gene function. Effective visualization of these isoforms and their expression patterns is crucial but challenging due to limitations in existing tools. Traditional genome browsers lack programmability, while other tools offer limited customization, produce static plots, or cannot simultaneously display structures and expression levels. RNApysoforms was developed to address these gaps by providing a Python-based package that enables concurrent visualization of RNA isoform structures and expression data. Leveraging plotly and polars libraries, it offers an interactive, customizable, and faster-rendering framework suitable for web applications, enhancing the analysis and dissemination of RNA isoform research.

**Availability and implementation:**

RNApysoforms is a Python package available at (https://github.com/UK-SBCoA-EbbertLab/RNApysoforms) and (https://zenodo.org/records/14941190) via an open-source MIT license. It can be easily installed using the pip package installer for Python. Thorough documentation and usage vignettes are available at: https://rna-pysoforms.readthedocs.io/en/latest/.

## 1 Introduction

Alternative splicing is a fundamental biological mechanism that enhances genetic diversity by allowing a single gene to produce multiple RNA isoforms. This process, occurring in over 95% of human multi-exon genes (Pan *et al.* 2008, Wang *et al.* 2008), results in mRNA variants that can have distinct and sometimes opposing functions. For instance, the *TRPM3* gene, which encodes human cation-selective channels, can be alternatively spliced to produce variants targeting different ions (Oberwinkler *et al.* 2005, Frühwald *et al.* 2012, Mittendorf *et al.* 2012). Similarly, *CASP3* has two transcript variants with opposing functions: one is pro-apoptotic, and the other is anti-apoptotic (Huang *et al.* 2001, Végran *et al.* 2006). Alternative splicing is intricately regulated, resulting in a rich diversity of context-dependent RNA transcripts emerging from the same genetic code.

Traditional RNA sequencing methods that rely on short-read technologies face significant challenges in accurately reconstructing and quantifying these RNA isoforms. Short reads often do not span multiple splice junctions, making it difficult to assemble full-length transcript structures (Conesa *et al.* 2016, Dong *et al.* 2023). This limitation can lead researchers to aggregate all RNA isoforms into a single gene-level measurement, potentially obscuring important biological insights. The recent improvements and increased affordability of long-read sequencing platforms, such as PacBio and Oxford Nanopore, has revolutionized transcriptomics by enabling the sequencing of full-length transcripts. These technologies facilitate the discovery of novel isoforms and provide more accurate quantification of RNA isoform expression, leading to new insights in human genomics (Leung *et al.* 2021, Glinos *et al.* 2022, Page *et al.* 2024), cancer (Zhou *et al.* 2022, Liu *et al.* 2023, Sun *et al.* 2023), Alzheimer’s disease (Heberle *et al.* 2024), and plant biology (Hu *et al.* 2022).

Visualizing RNA isoform structures and expression patterns is crucial for interpreting transcriptomic data and forming hypotheses about gene function. However, many existing visualization tools lack flexibility or do not effectively compare transcript structures. Genome browsers like UCSC (“UCSC Genome Browser Home,” 2024) and IGV (Robinson *et al.* 2023) facilitate transcript visualization but are not easily customizable through programming. Other tools, such as IsoformSwitchAnalyzeR (Vitting-Seerup and Sandelin 2019), and wiggleplotr (Alasoo 2024) offer certain functionalities but provide limited options for adjusting plot aesthetics and comparing transcript structures. While SWAN (Reese and Mortazavi 2021) offers customizable visualization within the Python environment, it has limitations in highlighting differences between isoforms and does not support interactive graphs.

Introduced in 2022, the ggtranscript (Gustavsson *et al.* 2022) R package provides a flexible and user-friendly framework for visualizing RNA isoform structures using ggplot2, a widely adopted R-based data visualization library. As a ggplot2 extension, ggtranscript allows extensive customization and integrates well with other ggplot2 functionalities. Despite its strengths, ggtranscript has notable limitations. It generates static plots without interactive capabilities, restricting users’ ability to dynamically explore RNA isoform structures—a significant drawback for web applications. Additionally, rendering complex plots can be time-consuming, making it less suitable for applications requiring rapid plot generation. Moreover, ggtranscript does not natively support concurrent visualization of RNA isoform expression levels alongside their structures, limiting its usefulness in studies focusing on differential isoform expression or usage.

To address these limitations, we developed RNApysoforms, a Python-based package designed to facilitate the simultaneous visualization of RNA isoform structures and expression levels. RNApysoforms aims to achieve three primary goals: (i) enable concurrent visualization of RNA isoform structures and their expression data; (ii) provide a flexible framework for creating interactive plots, allowing users to explore RNA isoform structures and expression in variety of useful ways; and (iii) enhance the dissemination of RNA isoform research findings through web applications by offering easy-to-use, interactive, customizable, and faster-rendering visualizations within the Python ecosystem. By leveraging the Python plotly (“Plotly Python Graphing Library,” 2012) and polars (“Polars,” 2024) libraries, RNApysoforms improves performance and user experience in web-based environments, making it a valuable tool for researchers in genomics, transcriptomics, and related fields.

## 2 Implementation

RNApysoforms is implemented as a modular Python package designed to streamline the analysis and visualization of RNA isoform data. At its core, the package leverages the high-performance polars library for rapid data manipulation and plotly for creating interactive visualizations. The design centers on several key functionalities that begin with robust data ingestion and validation. RNApysoforms supports the import of genomic annotations—most notably, ENSEMBL Gene Transfer Format (GTF) files—as well as expression matrices in various formats (TSV, CSV, XLSX, and Parquet). During this process, essential genomic features, such as exons and coding sequences (CDS), are identified, and any missing values are handled appropriately to ensure the integrity of the data.

In addition to data import, the package integrates expression matrices with sample metadata and provides normalization routines, including Counts Per Million (CPM) and relative transcript abundance calculations, to standardize the data for downstream analyses. Researchers can further refine their datasets by filtering genomic annotations and corresponding expression data to focus on specific genes; the package offers options to sort transcripts by their expression levels and select the most relevant isoforms. Recognizing the challenges inherent in visualizing complex transcript structures, RNApysoforms incorporates methods to enhance annotations. These methods compute exon numbering, derive intron coordinates from exon data, and rescale long intronic regions using approaches similar to those described in previous studies (Davidson *et al.* 2017, Gustavsson *et al.* 2022). Collectively, these techniques help produce more coherent and interpretable visualizations.

Finally, the package brings together these capabilities by assembling customized plotly traces and figures that integrate transcript structures with expression metrics. This interactive visualization framework allows users to explore and present their data with considerable flexibility, ensuring that complex transcriptomic information can be communicated effectively. Overall, RNApysoforms offers a comprehensive toolset that combines efficient data processing with advanced visualization, while also maintaining compatibility with common data formats and supporting interoperability with the pandas library through conversion to polars DataFrames.

## 3 Example use case

We demonstrate our package using an example RNA isoform expression dataset of eight samples—four Alzheimer’s cases and four controls. In this example, we generated a plot of the top five expressed *APP* RNA isoforms in our dataset. The *APP* gene encodes the amyloid precursor protein that forms amyloid plaques, a hallmark of Alzheimer’s disease. A complete vignette and the example dataset are available in the RNApysoforms documentation.

**Figure 1.**
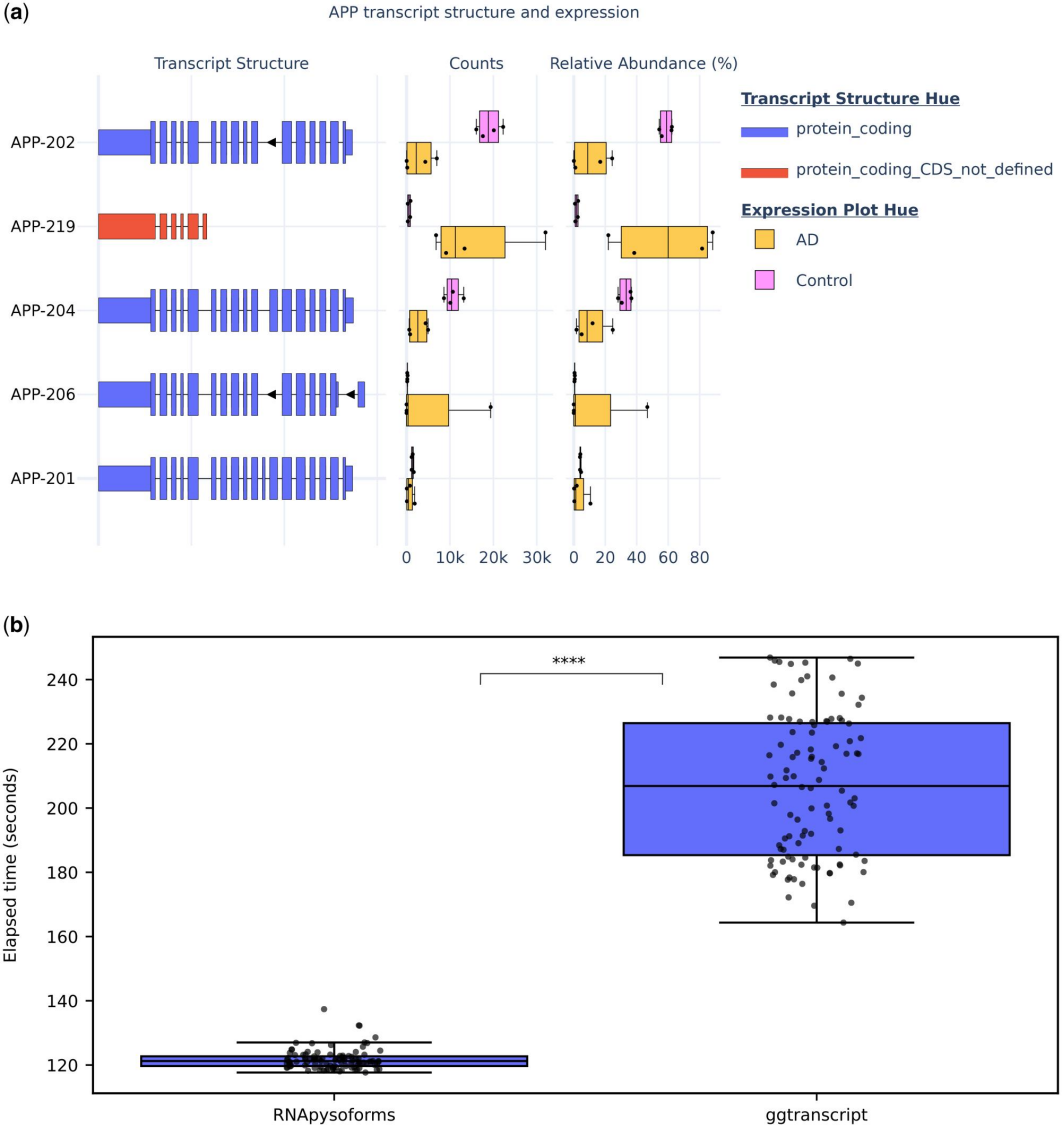
RNApysoforms visualization example and execution time comparison with ggtranscript. (a) Example plot generated by RNApysoforms illustrating the structure and expression of five APP RNA isoforms in a test dataset comparing Alzheimer’s disease (AD, *n*=4) with control (*n*=4) samples. An interactive version of the plot is available at the bottom of our vignette. (b) Boxplot showing the time taken by RNApysoforms and ggtranscript to render 1499 plots, encompassing all genes and transcripts from human chromosomes 21 and Y in ENSEMBL GTF release 107. Each package ran 100 iterations (*n*=100) with 1499 plots generated per loop. RNApysoforms achieved a median completion time of 121.25 s, 70% faster than ggtranscript’s median of 206.87 s. A permutation test on the median execution time difference yielded a significant *P*-value = 2 × 10^−7^, with a 95% bootstrap confidence interval for the execution time difference of [−94.59, −77.05] s. All boxplots in this panel follow the following format: median (center line), quartiles (box limits), 1.5 × interquartile range (whiskers). ****P-value < 0.0001.

Our vignette walks users through an end-to-end demonstration of RNApysoforms. It processes transcript count data and annotations to produce an interactive graph that displays both the structure and expression of the top five expressed APP isoforms. Given APP’s central role in Alzheimer’s disease, examining its RNA isoforms is critical; changes in their expression or structure can influence the production and processing of the amyloid precursor protein and, consequently, amyloid plaque formation. The static version of this figure (Fig. 1a) allows researchers to quickly identify expression patterns and inspect isoform structures—protein-coding regions are indicated as taller boxes in the “Transcript Structure” panel. Furthermore, RNApysoforms can be easily adapted to study RNA isoform expression for any gene or disease, making it a versatile tool for comprehensive transcriptomic analysis.

The interactive plot shown in our vignette further enhances data exploration. Users can zoom into specific regions and hover over elements—such as exons, introns, coding sequences, boxplots, and individual data points—to access detailed information. For example, hovering over an exon reveals its start and end coordinates, length, exon number, chromosome, and associated transcript. Additionally, the interactive legend enables users to toggle the data display by clicking on labels, offering another way to explore the dataset. With these features, RNApysoforms not only streamlines transcript isoform analysis across diverse genes and diseases but also provides a foundation for further development of dynamic, interactive web applications for in-depth RNA isoform exploration.

## 4 Execution time comparison

We evaluated the performance of RNApysoforms compared to ggtranscript by measuring the time required to render and save 1499 plots covering all genes and transcripts from human chromosomes 21 and Y using the ENSEMBL GTF release 107. Each software package was tested over 100 iterations (*n* = 100), with each iteration generating the complete set of plots. RNApysoforms saved plots in HTML format (natively supported by plotly), while ggtranscript used PDF format (natively supported by ggplot2). Due to differences in format and interactivity, RNApysoforms plots were significantly larger in size, occupying 6.5 GB for 1499 plots compared to ggtranscript’s 94 MB. Though, because RNApysoforms is typically rendered directly on the webpage, it does not generally need to generate output files. The execution time comparison scripts were run sequentially on the same compute node at the University of Kentucky’s Morgan Compute Cluster, utilizing 1 CPU and 4 GB of RAM. All data, code, outputs, and the software container required for this analysis are accessible via the GitHub repository here (https://github.com/UK-SBCoA-EbbertLab/article_analysis_RNApysoforms).

RNApysoforms demonstrated a median runtime of 121.25 s [interquartile range (IQR): 119.69–122.71 s], significantly outperforming ggtranscript’s median runtime of 206.87 s (IQR: 185.34–226.44 s). This substantial difference translates to RNApysoforms being approximately 70% faster, with a median time reduction of 85.63 s (Fig. 1b). Statistical analyses revealed significant deviations from normality in the runtime data for both tools, as indicated by Shapiro-Wilk tests (RNApysoforms: *W* = 0.826, *P* = 1.67 × 10^−9^; ggtranscript: *W* = 0.953, *P* = 0.0014), and unequal variances confirmed by Levene’s test (*F* = 238.66, *P* = 7.55 × 10^−36^). Consequently, we used a non-parametric permutation test on the median difference, which, using 10 million resamples, revealed a significant difference in execution times favoring RNApysoforms (*P* = 2 × 10^−7^). Although a smaller *P*-value might be obtained with more resamples, increasing the number of resamples was not practical due to the considerable computational resources required. To further quantify this advantage, we built a 95% bootstrap confidence for the difference in execution time using 10 million resamples and the interval was [−94.59, −77.05] s, underscoring RNApysoforms’ substantial performance edge. Furthermore, an effect size of −1.0 (Cliff’s delta) indicates a large and consistent performance difference. Collectively, these results establish RNApysoforms as a significantly faster tool for visualizing RNA isoform structures in large-scale genomic datasets.

## 5 Conclusion

RNApysoforms successfully addresses the limitations of existing RNA isoform visualization tools by providing a Python-based package that enables simultaneous visualization of RNA isoform structures and expression levels. By leveraging efficient data manipulation with polars and interactive plotting with plotly, it offers a flexible framework for creating customizable and interactive plots. Performance evaluations demonstrate that RNApysoforms significantly outperforms ggtranscript in rendering and saving plots, making it a faster and more efficient tool for large-scale genomic datasets and web applications. This enhanced performance, combined with its ease of use and integration into web applications, makes RNApysoforms a valuable resource for transcriptomics research.

## Data Availability

The data and code underlying this article are available in the RNApysoforms repository at https://github.com/UK-SBCoA-EbbertLab/RNApysoforms, https://github.com/UK-SBCoA-EbbertLab/article_analysis_RNApysoforms, https://zenodo.org/records/13961009, and https://zenodo.org/records/14941190.
